# Rotational alignment of the tibial component in total knee arthroplasty is better at the medial third of tibial tuberosity than at the medial border

**DOI:** 10.1186/1471-2474-11-57

**Published:** 2010-03-25

**Authors:** Jörg Lützner, Frank Krummenauer, Klaus-Peter Günther, Stephan Kirschner

**Affiliations:** 1 Department of Orthopaedic Surgery, University Hospital Carl Gustav Carus, Medical Faculty of the Technical University of Dresden, Dresden, Germany; 2 Clinical Epidemiology and Health Economy Unit, Department of Orthopaedic Surgery, University Hospital Carl Gustav Carus, Medical Faculty of the Technical University of Dresden, Dresden, Germany

## Abstract

**Background:**

Correct rotational alignment of the femoral and tibial component is an important factor for successful TKA. The transepicondylar axis is widely accepted as a reference for the femoral component. There is not a standard reference for the tibial component. CT scans were used in this study to measure which of 2 tibial landmarks most reliably reproduces a correct femoro-tibial rotational alignment in TKA.

**Methods:**

80 patients received a cemented, unconstrained, cruciate-retaining TKA with a rotating platform. CT scans were performed 5-7 days postoperatively but before discharge. The rotational mismatch between the femoral and tibial components was measured. Furthermore, the rotational variance between the transepicondylar line, as a reference for the orientation of the femoral component and different tibial landmarks, was measured.

**Results:**

There was notable rotational mismatch between the femoral and tibial components. The median mismatch was 0° (range: 16.2 degrees relative external to 14.4 degrees relative internal rotation of the femoral component).

Using the transepicondylar line as a reference for femoral rotational alignment and the medial third of the tuberosity as a reference for tibial rotational alignment, 67.5% of all TKA had a femoro-tibial variance within ± 5 degrees, 85% within ± 10 degrees and 97.5% within ± 20 degrees. Using the medial border of the tibial tubercle as a reference this variance was greater, only 3.8% had a femoro-tibial variance within ± 5 degrees, 15% within ± 10 degrees and 68.8% within ± 20 degrees.

**Conclusion:**

Using fixed bone landmarks for rotational alignment leads to a notable variance between femoral and tibial components. Referencing the tibial rotation on a line from the medial third of the tibial tubercle to the center of the tibial tray resulted in a better femoro-tibial rotational alignment than using the medial border of tibial tubercle as a landmark. Surgeons using fixed bearings with a high rotational constraint between the inlay and the femoral component should be aware of this effect to avoid premature polyethylene wear.

**Trial Registration:**

Clinical trials registry NCT01022099

## Background

The outcome of total knee arthroplasty (TKA) is dependant on multiple factors. In addition to patient-related factors, restoring the mechanical axis and balancing the soft tissue are important factors in obtaining proper rotational alignment of the femoral and tibial components. Rotational malalignment may lead to patellar maltracking, anterior knee pain, femoro-tibial flexion instability and premature wear of the polyethylene inlay [1-5]. Several studies have demonstrated higher revision rates and less favorable clinical results in patients with rotational malalignment of the femoral and tibial components [3,6,7]. In addition to these findings, a biomechanical study has demonstrated an increase in the tibial cortex strain at approximately 10° of rotational malalignment [8].

The transepicondylar axis is widely accepted as the best representation of the functional flexion-extension axis of the knee [4,9-13]. As a result, the transepicondylar line is used as a reference for the rotational alignment of the femoral component.

However, a standard reference for tibial rotational alignment remains controversial. Several different landmarks can be used for tibial rotational alignment including extraarticular references (i.e., the transmalleolar axis and the second metatarsal bone). Unfortunately, these references vary among patients and are therefore unreliable[14] Intraarticular references (i.e., the posterior tibial condylar line, the transcondylar tibial line or the line between the tibial spines) can be difficult to identify in osteoarthritic knees[14] Therefore, most surgeons use the tibial tubercle and the insertion of the posterior cruciate ligament on the posterior border of the tibia as reference points. These anatomic landmarks provide the most reliable landmarks for rotational alignment of the tibia[15] Three recommended points of the tibial tubercle that are used to determine the rotation of the component are the medial border, [14] the medial third [3] and the most prominent point of the tubercle [1,2]. Nonetheless, these landmarks vary greatly between patients [16-18].

Another option is the "self-seeking method", which aligns the tibial component according to the rotational alignment of the femoral component during trial reduction. This method induces the risk of transfering a femoral malrotation to the tibia [15].

We have previously reported our results of rotational alignment of the femoral and tibial component after navigated and conventional TKA using postoperative CT scans [19]. In this study we used these CT scans to measure femoro-tibial rotational mismatch in order to investigate which tibial landmark (the medial border or the medial third of the tibial tubercle) is the most reliable for a correct femoro-tibial rotational alignment. This investigation is based on the premise that rotational mismatch between foral and tibial component should be zero degree if both components are ideally implanted.

## Methods

The study protocol was approved by the local independent ethics committee of the Medical Faculty of the Technical University Dresden on 31.03.2005. All patients signed informed consent before study inclusion.

### Patients

80 patients with primary or secondary osteoarthritis of the knee, no previous hemi or total knee arthroplasty, a mechanical axis between 20° of varus and 5° of valgus and no severe instability received an unconstrained, cruciate-retaining TKA with a rotating platform during a prospective randomized study comparing navigated and conventional surgical technique [19]. There was no difference between the two surgical techniques for rotational alignment, varus/valgus alignment and demographic data and therefore the data from all 80 patients were pooled for further analysis of best tibial rotational alignment.

Due to the use of the rotating platform the femur and the tibia could rotate against each other as forced by the soft tissues. Therefore rotational deviation between femur and tibia could be measured. *Femoro-tibial rotational mismatch *was defined as the difference (in degrees) between femoral and tbial component on CT scans. "True" *femoro-tibial rotational variance *was defined as the difference (in degrees) between an ideal position of the femoral component (transepicondylar line) and a hypothesized ideal position of the tibial component (medial border or medial third of the tibial tubercle).

### Radiographic and CT evaluation

Five to seven days after the surgery, all patients received a radiographic and CT assessment. Radiographic evaluation of the leg axis and alignment of the components was performed as previously described [19]. The patellar tilt and the displacement of the patella were measured on a merchant view radiograph. The patellar tracking was defined as neutral if the tilt was ± 5° and the displacement less than 5 mm [20].

**Figure 1:**
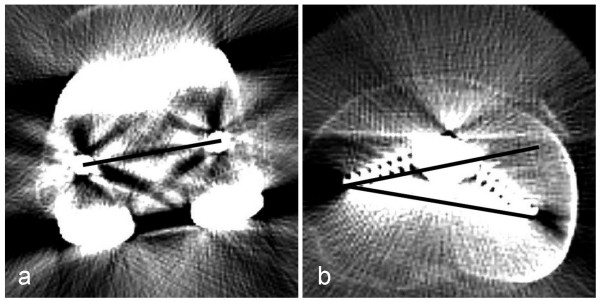
**Determination of rotational mismatch between femoral and tibial component**. a: Line through femoral fixation pegs for determination of the femoral component rotation, b: Angle between rotational alignment of the femoral and a line along the posterior border of the tibial component for determination of the tibial rotation.

The CT digital images were evaluated using the software ID.PACS Release 3.6 (Image Devices, Idstein, Germany). The rotational alignment of the femoral and tibial components were evaluated on the CT scans as already described[19] The rotational alignment of the femoral component was defined as a line through the centre of both femoral fixation pegs (Fig. 1a). The rotational alignment of the tibial component was defined as a line along the posterior border of the tibial stem. The rotational alignment of the femoral and tibial component were then superimposed and the mismatch between both was measured (Fig. 1b).

**Figure 2:**
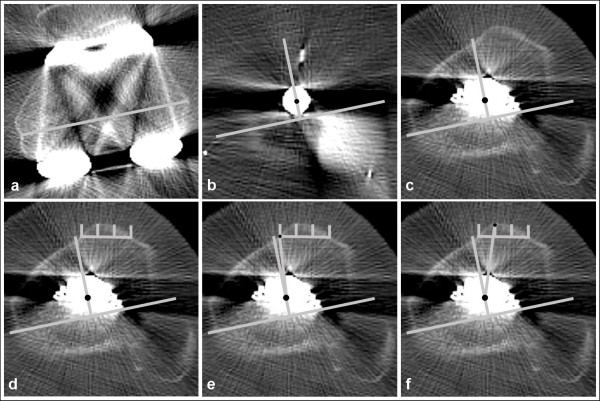
**Determination of the "true" rotational variance between femur and tibia**: Transepicondylar axis (a) is superimposed to the tibia and a perpendicular line is drawn through the rotational centre of the tibial tray (fixation peg for rotating platform, b). These lines are superimposed to a slice where the tibial tuberosity is visible (c). The tibial tuberosity is divided into three parts (d). The angle between a line from the medial border of the tibial tuberosity to the rotational center and the line perpendicular to the transepicondylar axis is measured (e). The angle between a line from the lateral border of the medial third of the tibial tuberosity to the rotational center and the line perpendicular to the transepicondylar axis is measured (f).

In order to obtain the "true" rotational variance between femur and tibia without any implantation failure, the transepicondylar axis (Fig. 2a) was superimposed to the tibia. From this line, a perpendicular line through the rotational center of the tibial tray (fixation peg for rotating platform, Fig. 2b) was drawn. These lines were superimposed on a slice where the tibial tuberosity was visible (Fig. 2c). The tibial tuberosity was divided into three parts (Fig. 2d). A line from the medial border of the tuberosity to the centre of the tibial tray was drawn. The angle between this line and the line perpendicular to the transepicondylar axis was measured (Fig. 2e). Additionally, a line from the lateral border of the medial third to the centre of the tibial tray was drawn. The angle between this line and the line perpendicular to the transepicondylar axis was also measured (Fig. 2f).

### Sample size considerations

The primary radiological endpoint of this investigation was the "true" rotational variance between the femur and the tibia, which was quantified by respectively using each of the two tibial alignment landmarks (the medial third or the medial border of the tuberosity). A deviation between femur and tibia of less than ± 10° was considered tolerable from a clinical perspective.

The sample size of the underlying controlled clinical trial was targeted for the comparison of tibial rotational alignment after navigated versus conventional TKA. However, the resulting total sample size of 80 patients can also be regarded sufficient for this investigation.

The confirmatory analysis of this investigation was based on a sign test, which was applied to intraindividually compare the "true" rotational variances' distributions when being assessed by the respective landmarks. The results of this confirmatory test were summarized in terms of a p-value.

To detect a median difference of at least 10° between the resulting mismatch distributions under assumption of a difference standard deviation of 20°, the effective sample size of 80 patients ensures a statistical power of 99%, when a two-sided paired t-test were applied at the 5% significance level. The paired sign test analysis, however, will not severely deviate from a formal t-test evaluation; the statistical power of the above confirmatory analysis concept will therefore certainely be at least 80%.

### Statistical analysis

Data description was based on medians and quartiles for continuous endpoints and on absolute and relative frequencies for categorical endpoints. Sub-sample comparisons were based on two sample Wilcoxon tests for continuous and on exact Fisher tests for categorical variables, respectively. Results of these exploratory significance tests were summarized in p-values, where p < 0.05 indicates locally significant differences between sub-samples. All analyses were performed according to the intention-to-treat principle by a certified medical biometrician (FK) using the SPSS^® ^software (SPSS Inc., Chicago, Illinois, USA, release 12.0 for Windows^®^).

## Results

### Rotational mismatch

The rotational mismatch in extension between femoral and tibial component was in median 0° (range: 16.2° relative external to 14.4° relative internal rotation of the femoral component). 71 TKA (89%) showed a femoro-tibial mismatch within ± 10° and 9 TKA (11%) outside of the ± 10° range.

### "True" femoro-tibial rotational variance

**Figure 3:**
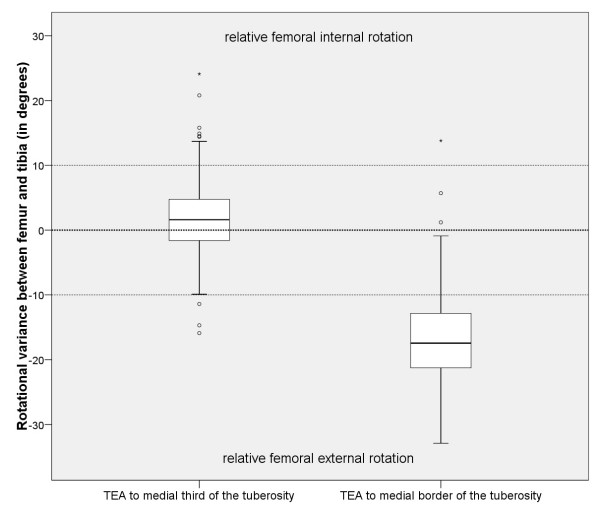
**"True" rotational variance between femur (transepicondylar axis) and different tibial landmarks (medial third or medial border of the tuberosity)**. (Negative values indicate femoral external rotation relative to the tibia, positive values indicate relative femoral internal rotation; Box plot horizontals indicate medians and quartiles, verticals indicate minumum and maximum observations. Circles and asterixes indicate statistical outliers).

The "true" rotational variance showed a statistically significant better femoro-tibial rotational alignment when referenced to the medial third of the tibial tubercle (p < 0.001, Fig. 3). The rotational deviation between the transepicondylar line and the medial border of the tuberosity was in median 17.4° relative external rotation of the femur (range: 32.9° relative external to 13.8° relative internal rotation of the femur). The deviation to the medial third of the tuberosity was in median 1.6° relative internal rotation of the femur (range: 15.9° relative external to 24.1° relative internal rotation of the femur).

Using the medial third of the tuberosity as a reference for tibial rotational alignment, 67.5% of all TKA had a "true" femoro-tibial variance within ± 5°, 85% within ± 10°, and 97.5% within ± 20°. Using the medial border of the tibial tubercle as a reference, the variance was greater with only 3.8% having a "true" femoro-tibial variance within ± 5°, 15% within ± 10° and 68.8% within ± 20° (Table 1).

**Table 1:** "True" rotational variance between femur (transepicondylar axis) and different tibial landmarks: medial border or medial third.

"true" rotational variance	medial border	medial third	
**median**	17.4°	-1.6°	

**range**	-13.8° to 32.9°	-24.1° to 15.9°	p < 0.001

**≤ ± 5°**	4%	68%	

**≤ ± 10°**	15%	85%	

**≤ ± 20°**	69%	98%	

Negative values indicate femoral external rotation relative to the tibia, positive values indicate relative femoral internal rotation.

The multivariate re-analysis of the mismatch between the femoral and tibial components as well as the "true" rotational variance did not reveal additional information as compared to the univariate analysis above. Multiple logistic regression modelling did not identify any clinical or radiographical cofactors as significantly associated with the primary endpoints. The assigned surgical treatment (navigated or conventional), the preoperative axis nor the BMI, sex or age of the patients did not have a statistically significant association with the primary endpoints.

### Patellar tracking

The patella was centred with a medio-lateral displacement of < 5 mm in all patients. The lateral tilt was in median 2.5° (range: 10.6° lateral to 2.7° medial tilt). Patellar tracking was neutral in 80% and showed a lateral tilt of > 5° in 20% of all patients. In the group with a tolerable mismatch (< ± 10°), 14 patients (20%) showed a lateral tilt of > 5° and 2 patients (22%) in the group with more than 10° of rotational mismatch showed a lateral tilt of > 5°.

## Discussion

Rotational malalignment has been demonstrated as an important cause of failure in total knee arthroplasty. While there is consensus about the transepicondylar axis as a reference for the femoral rotational alignment [4,9-13] there is no comparable agreement for the tibial rotational alignment.

To the author's knowledge, there has been no CT-controlled study performed to measure the variance between femoral and tibial component after TKA with correction of a femoral rotational malalignment.

Eckhoff et al. [16] implanted TKA in seven fresh frozen anatomic specimen knees and found as much as 19° of external rotation of the tibial component relative to the femoral component using the tibial tubercle as a reference for rotational alignment of the tibia. However, this study was performed on non-osteoarthritic knees.

Bindelglass et al. [21] measured intraoperatively the distance between the medial third of the tibial tubercle and the alignment of the tibial tray when seeking its own position during trial reduction. This resulted in 25 of 30 TKAs with an internally rotated tibial component relative to the tibial tubercle. It must be mentioned that a 3° of femoral external rotation relative to the posterior condylar line was used as a reference in the aforementioned study and not the transepicondylar axis. No correlation to the transepicondylar axis was performed. Therefore this relative internal rotation may be caused by a transferred femoral malrotation.

Incavo et al. [22] studied MRI scans of 30 normal knees. He templated an symmetric tibial baseplate using the femoral epicondylar axis as reference and measured the intersection of the midaxis line and the patellar ligament. This resulted in 22 of 30 knees with an optimal position of the tibial baseplate, defined as intersection of the midaxis line of the tibial template between the midpoint of the ligament and the midpoint of the medial third of the patellar ligament, which is consistent with our findings. However, this study was done on non-osteoarthritic knees and the patellar ligament was used as reference which may be difficult to obtain intraoperatively when everting the patella.

Akagi et al. [14] measured the angle between a line perpendicular to the transepicondylar axis and different landmarks on healthy subjects. They found the lowest variability in an axis from the medial border of the ligamentum patellae to the posterior cruciate ligament. However, this measurement was done on non-osteoarthritic knees and it is difficult to define the middle of the posterior cruciate ligament on CT scans after total knee arthroplasty. Furthermore the medial border of the ligamentum patellae could be difficult to identify after medial arthrotomy.

Uehara et al. [18] studied osteoarthritic knees with a varus malalignment before TKA. This study demonstrated a mean external rotation of 2,6° of the tibia relative to the transepicondylar axis (range: 16° external to 10° internal rotation) using a line from the medial third of the tibial tubercle to the midpoint of the longest medio-lateral distance.

Huddleston et al. [17] measured intraoperatively during trial reduction the deviation of the trial insert from the tibial tubercle. This angle was 5,2° external rotation (range: 10° internal to 15° external rotation) of the trial insert relative to the medial border of the tibial tubercle. For femoral rotation they used a mix of flexion-gap blancing, transepicondylar axis, Whiteside's line and posterior condylar line as reference points. No correlation to a possible femoral malrotation was done and the deviation was measured in steps of 5°.

Recently Cobb et al. [23] described the "anatomical tibial axis", which is the perpendicular to the mid-point of the line joining the medial and lateral condylar centres. This line was more reliable than the posterior condylar line or a line from the lateral tibial spine to the center of the tibial tubercle. However, this "anatomical axis" touches the tibial tubercle lateral of the medial border.

This investigation demonstrates that using an axis from the medial third of the tibial tubercle to the center of the tibial tray as a reference for the tibial rotational alignment leads to a better femoro-tibial alignment in extension than using the medial border of the tibial tubercle. This corresponds with the results of Huddleston [17] where the trial insert was rotated to a point 5° lateral to the medial border of the tibial tubercle, and results from Uehara [18] and Cobb's "anatomical axis" [23] which was lateral of the medial border of the tibial tuberosity.

While the deviation from the transepicondylar axis was low in our study group, there was a considerable range for the tibial rotational alignment as already demonstrated in previous studies [24,25]. This may be due to difficulties to define the correct landmarks on the tibial tubercle during surgery as well as during measurement. Because rotational alignment was calculated relative to the short axis (tibial tubercle to the centre of the tibial tray), a deviation of one millimeter at the tibial tubercle resulted in a rotational deviation of about five degrees (depending on the size of the tibia). Another limitation of this study is the retrospective design because the original aim was the precision of implant position in navigated and conventional TKA. However, the statistical power appeared to be sufficient to support the results.

However, it must be realized that there is a notable rotational variance between femoral and tibial component using a fixed bone landmark as a reference (even if both components are ideally implanted). This could possibly be compensated by using a rotating tibial platform or a prosthesis with a fixed bearing design, which allows a certain amount of rotational freedom between the femoral and the tibial component. Fixed bearing ultra-congruent inlays restrict the rotation between femoral and tibial component and must be critically discussed when using a fixed landmark for tibial rotational alignment. The rotational restriction of ultra-congruent inlays may result in premature polyethylene wear. Thus using the "self-seeking method" for rotational alignment of the tibial component could help prevent femoro-tibial mismatch. However, this induces the risk of transferring a femoral malrotation to the tibia and caution must be taken with the patellar tracking.

There was no difference in patellar tracking between patients with a "tolerable" rotational mismatch (± 10°) and patients with more than 10° of mismatch between the femoral and the tibial component. This might be due to the use of the rotating platform and could be different with a fixed bearing.

## Conclusion

Referencing the tibial rotation on a line from the medial third of the tibial tubercle to the center of the tibial tray resulted in a better femoro-tibial alignment than using the medial border of tibial tubercle as a landmark. Nonetheless, there is great variety in the rotational mismatch between the femoral and the tibial component using a fixed bone landmark (even if both components are ideally implanted). Surgeons using fixed bearing implants with a high rotational constraint between inlay and femoral component should be aware of this effect to avoid premature polyethylene wear.

## Competing interests

The authors declare that they have no competing interests, although this study was financially supported by Stryker Orthopaedics to cover the costs of the patient's insurance policies.

## Authors' contributions

JL, FK, KPG and SK designed the trial together. JL wrote the manuscript with contributions from FK, KPG and SK. FK was involved as statistician. JL and SK were participating actively in the recruitment of the patients. All authors have read and approved this manuscript.

## Pre-publication history

The pre-publication history for this paper can be accessed here:

http://www.biomedcentral.com/1471-2474/11/57/prepub

## References

[B1] Barrack RL Schrader T Bertot AJ Wolfe MW Myers L Component rotation and anterior knee pain after total knee arthroplasty Clin Orthop Relat Res 2001 392 46 10.1097/00003086-200111000-00006 11716424

[B2] Berger RA Crossett LS Jacobs JJ Rubash HE Malrotation causing patellofemoral complications after total knee arthroplasty Clin Orthop 1998 356 144 9917679 10.1097/00003086-199811000-00021

[B3] Hofmann S Romero J Roth-Schiffl E Albrecht T [Rotational malalignment of the components may cause chronic pain or early failure in total knee arthroplasty] Orthopade 2003 32 6 469 12819885 10.1007/s00132-003-0503-5

[B4] Insall JN Scuderi GR Komistek RD Math K Dennis DA Anderson DT Correlation between condylar lift-off and femoral component alignment Clin Orthop Relat Res 2002 403 143 10.1097/00003086-200210000-00022 12360020

[B5] Wasielewski RC Galante JO Leighty RM Natarajan RN Rosenberg AG Wear patterns on retrieved polyethylene tibial inserts and their relationship to technical considerations during total knee arthroplasty Clin Orthop Relat Res 1994 299 31 8119035

[B6] Romero J Stahelin T Binkert C Pfirrmann C Hodler J Kessler O The clinical consequences of flexion gap asymmetry in total knee arthroplasty J Arthroplasty 2007 22 2 235 10.1016/j.arth.2006.04.024 17275640

[B7] Incavo SJ Wild JJ Coughlin KM Beynnon BD Early Revision for Component Malrotation in Total Knee Arthroplasty Clin Orthop Relat Res 2007 458 131 6 17224835 10.1097/BLO.0b013e3180332d97

[B8] Kessler O Lacatusu E Sommers MB Mayr E Bottlang M Malrotation in total knee arthroplasty: Effect on tibial cortex strain captured by laser-based strain acquisition Clin Biomech (Bristol, Avon) 2006 21 6 603 9 10.1016/j.clinbiomech.2006.01.011 16554112

[B9] Asano T Akagi M Nakamura T The functional flexion-extension axis of the knee corresponds to the surgical epicondylar axis: in vivo analysis using a biplanar image-matching technique J Arthroplasty 2005 20 8 1060 10.1016/j.arth.2004.08.005 16376264

[B10] Churchill DL Incavo SJ Johnson CC Beynnon BD The transepicondylar axis approximates the optimal flexion axis of the knee Clin Orthop Relat Res 1998 356 111 10.1097/00003086-199811000-00016 9917674

[B11] Miller MC Berger RA Petrella AJ Karmas A Rubash HE Optimizing femoral component rotation in total knee arthroplasty Clin Orthop 2001 392 38 11716411 10.1097/00003086-200111000-00005

[B12] Olcott CW Scott RD The Ranawat Award. Femoral component rotation during total knee arthroplasty Clin Orthop Relat Res 1999 367 39 10546596

[B13] Olcott CW Scott RD A comparison of 4 intraoperative methods to determine femoral component rotation during total knee arthroplasty J Arthroplasty 2000 15 1 22 10.1016/S0883-5403(00)91051-9 10654458

[B14] Akagi M Mori S Nishimura S Nishimura A Asano T Hamanishi C Variability of extraarticular tibial rotation references for total knee arthroplasty Clin Orthop Relat Res 2005 436 172 10.1097/01.blo.0000160027.52481.32 15995437

[B15] Romero J Stahelin T Wyss T Hofmann S [Significance of axial rotation alignment of components of knee prostheses] Orthopade 2003 32 6 461 12819884 10.1007/s00132-003-0475-5

[B16] Eckhoff DG Metzger RG Vandewalle MV Malrotation associated with implant alignment technique in total knee arthroplasty Clin Orthop Relat Res 1995 321 28 7497681

[B17] Huddleston JI Scott RD Wimberley DW Determination of neutral tibial rotational alignment in rotating platform TKA Clin Orthop Relat Res 2005 440 101 10.1097/01.blo.0000185448.43622.77 16239791

[B18] Uehara K Kadoya Y Kobayashi A Ohashi H Yamano Y Bone anatomy and rotational alignment in total knee arthroplasty Clin Orthop 2002 402 196 12218484 10.1097/00003086-200209000-00018

[B19] Lützner J Krummenauer F Wolf C Günther K-P Kirschner S Computer-assisted and conventional total knee arthroplasty - A prospective, randomized study with radiographic and CT evaluations J Bone Joint Surg Br 2008 90-B 8 1039 10.1302/0301-620X.90B8.20553 18669959

[B20] Bindelglass DF Cohen JL Dorr LD Patellar tilt and subluxation in total knee arthroplasty. Relationship to pain, fixation, and design Clin Orthop Relat Res 1993 286 103 8425330

[B21] Bindelglass DF Rotational alignment of the tibial component in total knee arthroplasty Orthopedics 2001 24 11 1049 11727800 10.3928/0147-7447-20011101-13

[B22] Incavo SJ Coughlin KM Pappas C Beynnon BD Anatomic rotational relationships of the proximal tibia, distal femur, and patella: implications for rotational alignment in total knee arthroplasty J Arthroplasty 2003 18 5 643 10.1016/S0883-5403(03)00197-9 12934219

[B23] Cobb JP Dixon H Dandachli W Iranpour F The anatomical tibial axis - reliable rotational orientation in knee replacement J Bone Joint Surg Br 2008 90 8 1032 10.1302/0301-620X.90B8.19905 18669958

[B24] Matziolis G Krocker D Weiss U Tohtz S Perka C A prospective, randomized study of computer-assisted and conventional total knee arthroplasty. Three-dimensional evaluation of implant alignment and rotation J Bone Joint Surg Am 2007 89 2 236 10.2106/JBJS.F.00386 17272435

[B25] Chauhan SK Scott RG Breidahl W Beaver RJ Computer-assisted knee arthroplasty versus a conventional jig-based technique. A randomised, prospective trial J Bone Joint Surg Br 2004 86 3 372 10.1302/0301-620X.86B3.14643 15125124

